# Counting on the mental number line to make a move: sensorimotor (‘pen’) control and numerical processing

**DOI:** 10.1007/s00221-017-5019-z

**Published:** 2017-07-27

**Authors:** Rebecca Sheridan, Maaike van Rooijen, Oscar Giles, Faisal Mushtaq, Bert Steenbergen, Mark Mon-Williams, Amanda Waterman

**Affiliations:** 1 School of Psychology, University of Leeds, Leeds, YSW LS2 9JT UK; 2 Behavioural Science Institute, Radboud University Nijmegen, Nijmegen, The Netherlands; 3 School of Psychology, Australian Catholic University, Melbourne, Australia

**Keywords:** Sensorimotor control, Motor, Movement, Number, Maths

## Abstract

Mathematics is often conducted with a writing implement. But is there a relationship between numerical processing and sensorimotor ‘pen’ control? We asked participants to move a stylus so it crossed an unmarked line at a location specified by a symbolic number (1–9), where number colour indicated whether the line ran left–right (‘normal’) or vice versa (‘reversed’). The task could be simplified through the use of a ‘mental number line’ (MNL). Many modern societies use number lines in mathematical education and the brain’s representation of number appears to follow a culturally determined spatial organisation (so better task performance is associated with this culturally normal orientation—the MNL effect). Participants (counter-balanced) completed two consistent blocks of trials, ‘normal’ and ‘reversed’, followed by a mixed block where line direction varied randomly. Experiment 1 established that the MNL effect was robust, and showed that the cognitive load associated with reversing the MNL not only affected response selection but also the actual movement execution (indexed by duration) within the mixed trials. Experiment 2 showed that an individual’s motor abilities predicted performance in the difficult (mixed) condition but not the easier blocks. These results suggest that numerical processing is not isolated from motor capabilities—a finding with applied consequences.

## Introduction

Mathematics is one of the pinnacles of human endeavour, raising phylogenetic and ontogenetic questions about the origins of this ability. It has been argued that many species have an innate sense of number (‘numerosity’) providing an animal with an approximate sense of quantity (Dehaene 1997). The presence of numerosity in humanoid ancestors presumably allowed the evolution of mathematical skills in humans. The evolution of language skills subsequently would have provided humans with additional ‘cognitive tools’ capable of supporting the development of numerical concepts. Nevertheless, the high levels of mathematical literacy found in many adults exist only because of cultural transmission across geography and time via educational systems (we define mathematical literacy as the ability to use and engage with mathematics in ways that meet an individual’s needs). The transmission of mathematics over time has produced increasingly sophisticated and abstract operations (e.g., arithmetic leading to algebra).

In this regard, the ontogeny of mathematical ability recapitulates phylogeny, with children first mastering basic arithmetic before learning increasingly abstract mathematical operations (Piaget and Garcia 1983). This pattern has been proposed to occur because abstract concepts (e.g., number and time) arise from sensorimotor interactions with a spatial world (Piaget and Garcia 1983). Indeed, evidence suggests that spatial processing abilities in the first year of life can predict mathematics ability later in early childhood (Lauer and Lourenco 2016). This hypothesis has physiological support from the presence of anatomically intermingled neurons in the prefrontal cortex and the fundus of the intraparietal sulcus, encoding length, numerosity, or both quantities in non-human primates (Tudusciuc and Nieder 2009). More generally, representations of number and space appear to share overlapping neural circuitry in the parietal lobes (Chen and Verguts 2010; Hubbard et al. 2005). The overlapping circuitry is consistent with evidence that number representation is spatially organised in the adult brain (Dehaene et al. 1993; Gobel et al. 2011; Hubbard et al. 2005; Marghetis et al. 2014; Sullivan et al. 2011). The most commonly reported behavioural evidence is the SNARC effect whereby Western educated individuals respond to smaller numbers faster when the response is on the left side and vice versa (Dehaene et al. 1993), with the direction of the SNARC effect being influenced by an individual’s culture (Dehaene et al. 1993; Fischer et al. 2010; Gobel et al. 2011; Hubbard et al. 2005; Fischer and Shaki 2014). There is some debate regarding the nature of these spatial–number associations. Gevers et al. (2003a, b) have demonstrated that letters of the alphabet and months of the year are also associated with spatial responses. Furthermore, van Dijk and Fias (2011) showed that the SNARC effect depends on the ordinal position of numbers in working memory rather than numerical size (see also Lindemann et al. 2007). These findings have led some authors to argue for the coexistence of separate mechanisms for ‘magnitude’ and ‘ordinality’ processing (Chen et al. 2014), and the relation of magnitude and ordinality to space (Huber et al. 2016; Prpic et al. 2016). In support of these arguments, Krause et al. (2014) have demonstrated structural brain differences related to the mapping of numbers to space and the mapping of numbers to sensorimotor-related magnitude.

However, the debates about the nature of the spatial–number associations do not impact on the overwhelming evidence showing that numbers are represented in a manner that maps to the physical world. In support of this idea, Wilson (2010) has argued that the representation and manipulation of numbers is related to the physical systems used to conduct mathematical operations within an individual’s culture. For example, counting systems that use different body parts (the fingers versus the spaces between the fingers) result in different base systems (base 10 versus base 8) and numeral representations (Selin 2001). Furthermore, reading, writing and finger counting typically follow a left to right direction in Western cultures and the motor elements of these behaviours may explain why numbers are represented in a culturally specific manner (Fischer and Brugger 2011). Indeed, this is exploited in modern teaching methods where ‘number lines’ have been regularly used for teaching mathematics to young children since the 1950s (Heeffer 2011). Number lines make the relationship between number and spatial location explicit and this may encourage the development of a ‘cognitive tool’—a ‘mental number line’ (MNL)—that facilitates numerical calculations.

There are a number of studies demonstrating that spatial representations (e.g., the MNL) are used as cognitive tools in maths tasks (e.g. counting). For example, Klein et al. (2014) used eye movements and estimation errors to show that mental addition on a number line is associated with a rightwards spatial bias whilst subtraction is associated with a leftward bias. Göbel (2015) found that counting is influenced by cultural norms of reading direction. Hartmann et al. (2016) used eye movements to show that space is used to operate on numbers in tasks such as counting. Mathieu et al. (2016) showed that calculations involving addition were solved faster when the second operand (sequentially displayed) was shown on the right, but subtraction calculations were faster when the second operand was on the left. Notably they found no bias when the second operand was zero or the operator was a multiplication. They concluded that a sequential mental representation of numbers is elicited during single-digit arithmetic.

More generally, there is a body of literature that suggests numerical processing is intrinsically linked with a variety of sensorimotor processes (e.g. Besner and Coltheart 1979; Henik and Tzelgov 1982; Cohen Kadosh and Henik 2006; Badets et al. 2007; Domahs et al. 2010; Link et al. 2013; Krause et al. 2013; Krause et al. 2016; Fisher 2012; Moeller et al 2012). On the basis of this work, we hypothesised that the sensorimotor control processes involved in writing (with a pen, pencil, piece of chalk or tablet stylus) would be intrinsically intertwined with the ‘higher-order’ cognitive processes involved in numerical processing (and more generally in any cognitive processing that relies on spatial representations). This viewpoint runs contrary to traditional views of human behaviour where cognitive processes are seen as being somewhat encapsulated from ‘lower-level’ motor output systems, but our notion has some support from a study by Fisher (2003) who reported that the kinematics of pointing movements were subject to a SNARC effect. On this basis, we hypothesised that not only would movement planning to a physical number line be influenced by the SNARC effect, but the resulting movement execution would also be affected. We anticipated that the effect would be greater when the cognitive load was higher (as the interference would be larger). If it is the case that movement execution is affected by the SNARC effect then this leads to the consequent hypothesis that individuals with better motor ability will show less interference (thus better performance) on a task that requires flexibility in the orientation of the mental number line.

We directly tested our hypotheses by developing a task where participants were asked to move a handheld stylus to cross a number line at a position indicated by an Arabic numeral. The use of lines to explore numerical representation is a well-established technique (e.g., Siegler and Opfer 2003). The colour of the symbol indicated whether the line ran left to right or right to left. Thus, the participant needed to determine the line direction (indicated by the colour), compute the quantity (indicated by the symbol) and then move the hand to the specified location. Thus, the brain could, in principle, carry out an abstract computation and the time taken to determine the target location would then be the same regardless of line direction. Conversely, the system could directly map a mental number line to the physical line. This process might be facilitated if participants utilised a default mental number line (MNL). A default MNL would result in faster responses when the physical number line was orientated in a direction consistent with the mental spatial–numerical representation (e.g., a representation established through cultural norms) and vice versa (the ‘MNL effect’). We were particularly interested in exploring whether the MNL effects would be found in the execution of the movements after the response had been selected, whether this increased with cognitive load, and whether this meant that individuals with superior motor abilities showed less interference. Some studies have investigated the impact of number processing on hand movements using mouse tracking (e.g., Faulkenberry 2016; Fischer and Hartmann 2014). However, Faulkenberry (2016) did not investigate kinematics per se, and Fischer and Hartmann (2014) discuss in their paper the problems associated with using mouse tracking to obtain kinematics. We used a high resolution system capable of providing precise end point kinematics, and therefore the two experiments reported here constitute the first detailed investigation into the kinematics of movements made under different cognitive demands (relating to numerical processing), and how sensorimotor control abilities relate to performance on a numerical processing task.

## Experiment 1

We designed a number line task in which participants rapidly selected the appropriate action in response to an imperative stimulus. This included ‘consistent’ blocks of trials where the physical number line was presented in the same orientation across all trials in that block. That is, all trials presented the number line *either* representing small numbers on the left (consistent ‘normal’ block), *or* representing small numbers on the right (consistent ‘reversed’ block). The ‘mixed’ condition presented trials where line direction varied randomly between normal and reversed. The mixed block was immediately preceded either by a consistent ‘normal’ block’, or by a consistent ‘reversed’ block. Therefore, the first group of participants completed the normal block first, then the reversed block, followed by the mixed block, whilst the second group of participants completed the reversed block first, then the normal block, followed by the mixed block. We measured participant’s reaction times which are taken to reflect the cognitive processes associated with movement planning, and movement times which represent movement execution (Fischer 2003).

### Method

#### Participants

Thirty-nine adults (14 males, mean age 26.4 years, range 18.3–56.8 years) participated in the study, with 20 participants in condition one (8 males, mean age 26.9 years, range 22–33 years, *SD* = 3.69) and 19 in condition two (6 males, mean age 25.6 years, range 18.3–56.8 years, *SD* = 10.64). We started with 46 participants but eight participants were removed from the experiment as they did not complete at least 50% of the trials correctly in the trial blocks. The majority of participants were right handed (*n* = 34; self-reported) and all spoke English as their first language (reading and writing words from left to right). The participants were University educated and had completed courses in statistics and were therefore reasonable ‘mathematically literate’. Participants in the two conditions did not differ in age, *F*(1, 38) = .24, *p* = .627, gender, χ2(1, *N* = 39) = .30, *p* = .584, or handedness, χ2(1, *N* = 39) = .17, *p* = 1.00. Ethical approval for the study was obtained from the University Ethics committee and the research was carried out in accordance with the provisions of the World Medical Association Declaration of Helsinki.

#### Materials

The experimental task was deployed on a touch screen tablet PC (Toshiba Portege M700-13P, 257 × 160 mm, 1280 × 800 resolution, 100 Hz refresh rate). The task was designed on the Kinematic Assessment Tool software (Culmer et al. 2009; Flatters et al. 2014a), using the LabView development environment (Version 8.2.1, National Instruments, Austin, TX). The laptop screen was folded back to provide a horizontal surface, which could be interfaced using a stylus as an input device (sampled at a 120 Hz).

**Fig. 1:**
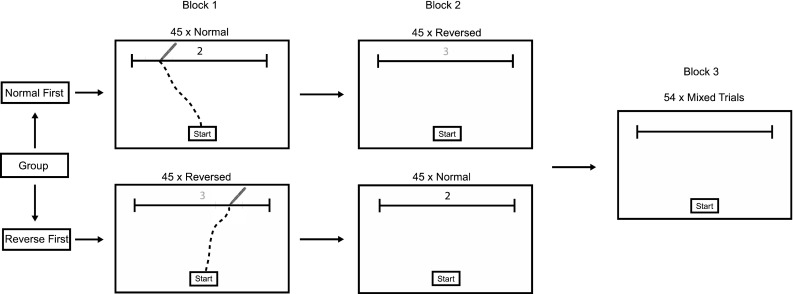
The experimental set up showing the procedure for each condition. Examples reflect participant data

The task involved participants moving from a start location shown on the screen to the appropriate location on a horizontal line 110 mm from the start location (see Fig. 1). The target location was indicated by a number shown above the line (the number was located above the centre of the line). Participants were told that the end of the line represented the numbers 0 and 10 and the line itself contained the numbers 1–9 equally spaced along the line. Participants were instructed that the number line ran left to right when the number was shown in red and ran right to left when the number was shown in blue. For the trials in the mixed block, participants were told that line direction would change randomly. Participants learned the colour to line direction correspondence in the consistent trials and were thus primed by number colour in the mixed trials. All participants confirmed that they readily understood the instructions.

Participants were instructed to hold the pen in their preferred hand and move it across the screen surface as fast as possible after the imperative number appeared (500 ms after the participant moved into the start box). They were instructed to slide the pen to fully cross the line where they thought the number belonged. Participants were seated at a comfortable position in front of the computer, approximately 400–600 mm away.

#### Procedure

All participants undertook three blocks of trials, with the order of the first two blocks counterbalanced across participants. Participants in condition A completed the normal block first, then the reversed block and then the mixed block. Participants in condition B completed the reversed block first, then the normal block and then the mixed block. The normal block consisted of 45 trials, the reversed block consisted of 45 trials and the mixed block consisted of 54 trials.

When participants were ready to begin the task, they held the stylus on the start button which triggered a number between 1 and 9 to appear above the line. The numbers were generated in a pseudorandom order; the correct response could not be on the same side of the screen more than three times in a row and the same number could not be presented consecutively. The number was red when the line ran from 0 to 10 (normal), and blue when it was from 10 to 0 (reversed). Participants used this information to determine line direction in the mixed block and were tasked with instructions to respond as quickly and as accurately as possible.

The CKAT software generated (i) reaction times (RTs; the time taken from the appearance of the imperative stimulus to the time the stylus moved from the start box) and (ii) movement times (MTs; the time taken between the stylus exiting the start box and crossing the number line). The movement of the stylus was thus determined from its spatial location on the screen rather than its change in velocity. It is possible to measure spatial inaccuracy to within 1 mm with the CKAT system (see Culmer et al. 2009). We were, therefore, able to measure the distance between where the stylus crossed the line and the veridical location specified by the number. All data were processed using MATLAB R2010a. We removed trials where the participant crossed the line on the wrong side (1.53% of trials). We also excluded trials (5.3%) if they had negative RTs (i.e. moved before line onset) and/or had movement times longer than 10 s.

## Results

### Data analysis

Reaction time data and movement time data were used to determine the presence of MNL effects. This was analysed by condition (whether participants completed the normal block or the reversed block first) and by trial type. All participants completed four trial types; normal trials from the consistent block (hereafter referred to as ‘normal’), reversed trials from the consistent block (hereafter referred to as ‘reversed’); and normal and reversed trials from the mixed block (‘mixed normal’ and ‘mixed reversed’). Partial eta squared effect sizes are reported (Cohen 1988) and the Greenhouse-Geisser correction applied where appropriate.

### Reaction times

**Fig. 2:**
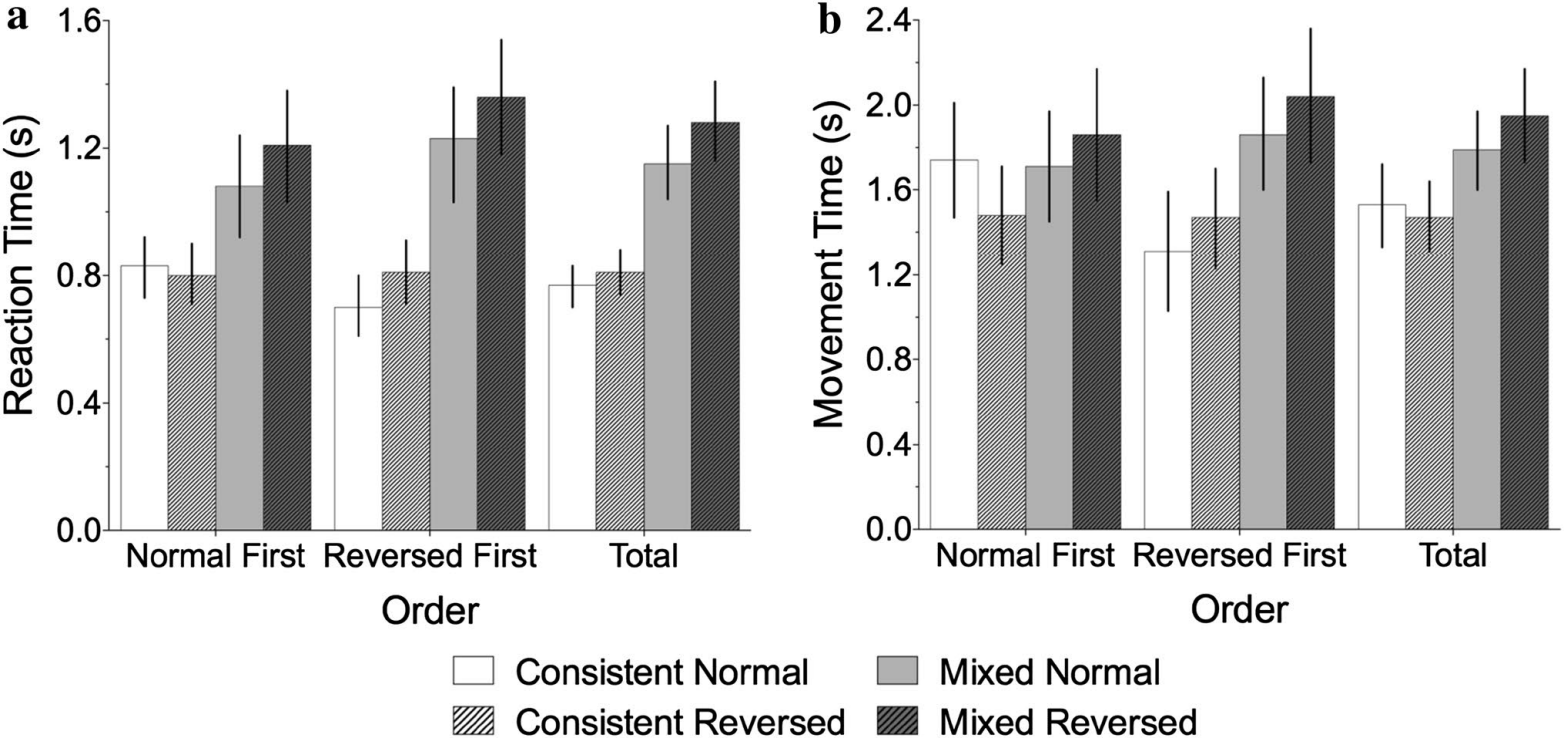
Reaction time (**a**) and movement time (**b**) data from Experiment 1

We first explored the effect of Condition (2 between participant levels: Normal First, Reversed First) and trial type (4 within participant levels: consistent normal, consistent reversed, mixed normal and mixed reversed) using a mixed-design ANOVA. A main effect of trial type was found, *F*(3, 111) = 81.23, *p* < .001, *η*_*p*_^*2*^ = .69. Overall, mixed trials showed slower reaction times than consistent trials. There was no main effect of condition, *F*(1, 37) = .33, *p* = .571, *η*_*p*_^*2*^ = .01, but a significant interaction, *F*(3, 111) = 5.32, *p* < .05, *η*_*p*_^*2*^ = .13. These effects can be seen in Fig. 2.

This interaction found in the ‘omnibus’ ANOVA was explored by doing separate analyses for the consistent blocks and the mixed block. First, in the consistent blocks there was no main effect of condition, *F*(1, 37) = .76, *p* = .39, *η*_*p*_^*2*^ = .02, but there was a main effect of trial type, *F*(1, 37) = 5.63, *p* = .023, *η*_*p*_^*2*^ = .13, with normal trials quicker than reversed trials. There was also an interaction between trial type and condition, *F*(1, 37) = 14.64, *p* < .001, *η*_*p*_^*2*^ = .28 where the MNL effect (i.e., normal trials quicker than reversed trials) was only found in the Reversed First condition (see Fig. 2a). This differential presence of a MNL effect depending on condition may reflect a small element of task learning at the start of the experiment. The learning period would be expected to marginally lengthen RTs. Therefore, when ‘normal’ trials were completed first this may have off-set the advantage gained from a MNL.

Second, for the mixed block, again there was no main effect of condition, *F*(1, 37) = 1.73, *p* = .196, *η*_*p*_^*2*^ = .05, but a main effect of trial type, *F*(1, 37) = 22.80, *p* < .001, *η*_*p*_^*2*^ = .38. This time the interaction was not significant, *F*(1, 37) = .00, *p* = .950, *η*_*p*_^*2*^ = .00: normal trials were responded to faster than reversed trials, regardless of the preceding block. Therefore, under high-difficulty conditions in the mixed block, there was a stronger effect of trial type (*η*_*p*_^*2*^ = .38, compared with *η*_*p*_^*2*^ = .13 in the consistent blocks), with RTs to normal trials faster than to reversed trials. In addition, the preceding block made no difference to this preference for a culturally orientated MNL.

### Movement times

As with the RT data, we first explored the effect of Condition (2 between participant levels: Normal First, Reversed First) and trial type (4 within participant levels: consistent normal, consistent reversed, mixed normal and mixed reversed) using a mixed model ANOVA. A main effect of trial type was found, *F*(3, 111) = 32.99, *p* < .001, *η*_*p*_^*2*^ = .47 (mixed blocks showed slower movement times than consistent blocks). There was no main effect of condition, *F*(1, 37) = .02, *p* = .88, *η*_*p*_^*2*^ = .001, but a significant interaction, *F*(3, 111) = 12.70, *p* < .001, *η*_*p*_^*2*^ = .25 (see Fig. 2b).

This interaction was further explored by running separate analyses for the consistent and mixed blocks. For the consistent blocks, there was no effect of trial type, *F*(1, 37) = 1.348, *p* = .253, *η*_*p*_^*2*^ = .035: there was no difference in MTs for normal vs reversed trials. However, for the mixed block the effect of trial type was significant, *F*(1, 37) = 23.43, *p* < .01, *η*_*p*_^*2*^ = .388: as with the RT data, normal trials showed shorter durations than reversed trials, regardless of the preceding block. None of the interactions were significant (*F*s < 1.0)

### Distance errors

**Fig. 3:**
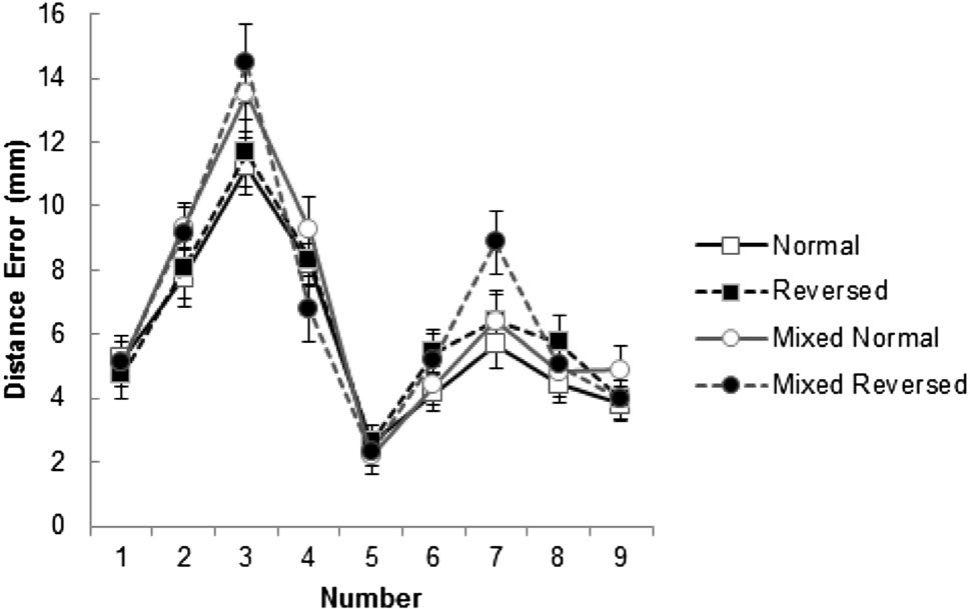
Average unsigned distance error for each number by trial type. *Error bars* represent ± 1 standard error of the mean. Data points are connected by lines to highlight the pattern of results, not due to the nature of the data

We next explored the effect of number on the distance error. We removed trials where the participant crossed the line on the wrong side (1.53% of trials). Significantly fewer errors were made in the consistent trials than in the mixed trials, *t*(38) = −5.01, *p* < .001, but no other influences on these errors reached significance. The participants were accurate when moving to the middle of the line (‘number 5’), and showed high precision (being within a few millimetres to the left or right of the line centre. The participants were also reasonably accurate when moving to the far extremes (numbers 1 and 9) but showed a bias towards the centre. Conversely, participants showed a bias away from the centre when moving towards numbers 4 and 6. The participants lacked precision (see Fig. 3) when moving towards numbers 3 and 7 but showed no systematic biases (i.e., they were accurate on average when moving to these numbers). These responses make sense as the number line was bounded by the vertical end stop and the centre with these locations providing clear ‘landmarks’. There was increasing uncertainty for numbers away from the centre and this can explain why the data showed a contraction bias (a tendency for the responses to be biased towards the mean of the range—i.e., the numbers 3 and 7). Figure 3 shows the unsigned distance error as a function of target number and illustrates the decreasing precision away from the end and centre of the line. The unsigned distance error was calculated by assigning a positive value to the error whether it was to the left or right of the veridical location.

Average distance errors were collapsed into two groups within trial types; small (numbers 1–4) and large (numbers 6–9). A repeated measures ANOVA with 8 levels revealed a significant effect of number type, *F*(7, 266) = 23.02, *p* < .001, *η*_*p*_^*2*^ = .38. This was due to bigger distance errors being observed for small numbers than large numbers in all trial types (normal, reversed, mixed normal and mixed reversed; all *p’s* < .001) as can be seen in Table 1.

**Table 1:** Distance error by trial type and number type in millimetres [95% confidence interval]

	Normal	Reversed	Mixed normal	Mixed reversed
1–4	8.11 [6.88, 9.34]	8.19 [7.11, 9.27]	9.31 [8.03, 10.59]	8.88 [7.47, 10.30]
6–9	4.53 [3.65, 5.42]	5.12 [4.11, 6.12]	5.13 [4.13, 6.14]	5.78 [4.75, 6.81]

One possible reason for the differences in movement time is that the participants showed different spatial accuracy according to the task demand (i.e., there was a speed accuracy trade-off in operation). To check for this possibility, average distance error was explored by trial type (4 within participant levels: normal, reversed, mixed normal and mixed reversed) and condition (2 group levels: Normal First and Reversed First) using a mixed-design ANOVA. No main effects of either trial type, *F*(3, 111) = 3.04, *p* > .05, *η*_*p*_^*2*^ = .08, or condition, *F*(1, 37) = .32, *p* = .576, *η*_*p*_^*2*^ = .01, were observed and there was no interaction, *F*(3, 111) = 1.83, *p* = .166, *η*_*p*_^*2*^ = .05. Thus, the differences in movement time could not be explained by differences in spatial accuracy.

An anonymous reviewer suggested that we explore the extent to which the task affected both the cognitive operations and the movement execution by correlating the reaction time and movement time data across the participants. We found a significant correlation between the average reaction time and movement time within the mixed trials, *r*(39) = .800, *p* < .001.

## Discussion

The results of Experiment 1 demonstrate that adults exploit a default number line representation under high-difficulty conditions (the mixed block). Notably, this default representation was consistent with long-term cultural norms and was not affected by exposing adults to a reversed number line immediately before. With regard to the less difficult (consistent) blocks, there was some evidence for the use of a MNL in the RT data, but this effect was smaller and less robust (with possibly some off-setting of the MNL advantage by task learning effects).

We also found evidence of the MNL affecting the movement itself (indexed by differences in the MT data), with adults moving more quickly to a ‘normal’ vs. ‘reversed’ orientation under higher difficulty conditions (the mixed block). In the number line task, the cognitive processing took longer when the required response was not consistent with the default cultural organisation of numerals. The impact of the cognitive processing was seen in both the reaction time and movement time data (and the two measures were highly correlated). If cognition were a closed system there would be no reason to suppose that there would be any impact of the MNL once the spatial position has been determined. However, the results from Experiment 1 suggest that the cognitive processes do affect motor execution. Indeed, this is consistent with a body of neurophysiological work which demonstrates joint recruitment of neural structures for motor and cognitive tasks. For example, the dorsolateral prefrontal cortex (primarily thought of as a cognitive structure) and the neo-cerebellum (primarily a motor structure) both show increased activation during cognitive tasks and decreased activation during well learned motor tasks (see Diamond 2000, for review). Furthermore, motor and cognitive deficits frequently co-occur in children. For example, developmental coordination disorder is often coupled with learning difficulties in tasks such as mathematics (Pieters et al. 2012). Likewise, children with cerebral palsy appear to have difficulties with mathematics in a manner that is not readily explained by cognitive deficit, but does appear to be related to the child’s motor ability (Van Rooijen et al. 2011). Finally, Simms et al. (2016) have recently shown that mathematical achievement can be explained by visuomotor integration and visuospatial skill competency.

Given the inter-dependency between cognition and action, we might expect that motor skill would influence performance on the task. In particular, when the cognitive demands of the task are increased (as in the mixed block) and the cognitive-motor system is therefore put under pressure, less proficient motor ability might have a deleterious effect on task performance. Experiment 2 sought to replicate the results of the first experiment, and also investigate whether motor ability is related to numerical processing performance.

## Experiment 2

Experiment 2 included measurements of performance on a simple aiming task where the cognitive demands were minimised (as the task only required movements to a physically specified target displayed on the tablet computer screen). The aiming task did not require the manipulation of symbolic information or the memory of the target location. Performance on the aiming task (measured as the average time to move between presented targets) serves as a proxy for motor skill (critically, the relevant motor skill required in the number line task) and has been shown to improve with increasing age over childhood and in line with improvements on other motor tasks (Flatters et al. 2014a, b). Under relatively low difficulty conditions (consistent blocks), adults’ motor skill may not play as important a role in completing the experimental task. However, when the cognitive-motor system is put under pressure (mixed blocks) motor skill may become increasingly important. Therefore, we hypothesised that performance in the consistent blocks would not be related to performance on the aiming task, but that there would be a relationship between performance on the aiming task and the mixed block.

### Method

#### Participants

Forty-seven adults took part in this study (23 females, age mean 22.3 years, range 20.5–47.7 years, SD = 3.88). We started with 54 participants but seven participants were removed from the experiment as they did not complete at least 50% of the trials correctly in the trial blocks. The participants were University educated and had completed courses in statistics and were therefore reasonable ‘mathematically literate’. Consistent with Experiment 1, the order in which participants completed the task was counterbalanced with 26 participants in condition one and 21 participants in condition two. Thirty-six participants were right handed and all participants spoke English as their first language. Participants in the two conditions did not differ by age, *F*(1, 46) = 1.67, *p* = .203, *η*_*p*_^*2*^ = .04, gender, χ2(1, *N* = 48) = .03, *p* = .858, or handedness, χ2(1, *N* = 48) = .15, *p* = .696. Ethical approval for the study was obtained from the University Ethics committee and the research was carried out in accordance with the provisions of the World Medical Association Declaration of Helsinki.

#### Materials

Materials for the number line task were identical to those used in Experiment 1. For the aiming task, the same tablet computers were used.

#### Procedure

**Fig. 4:**
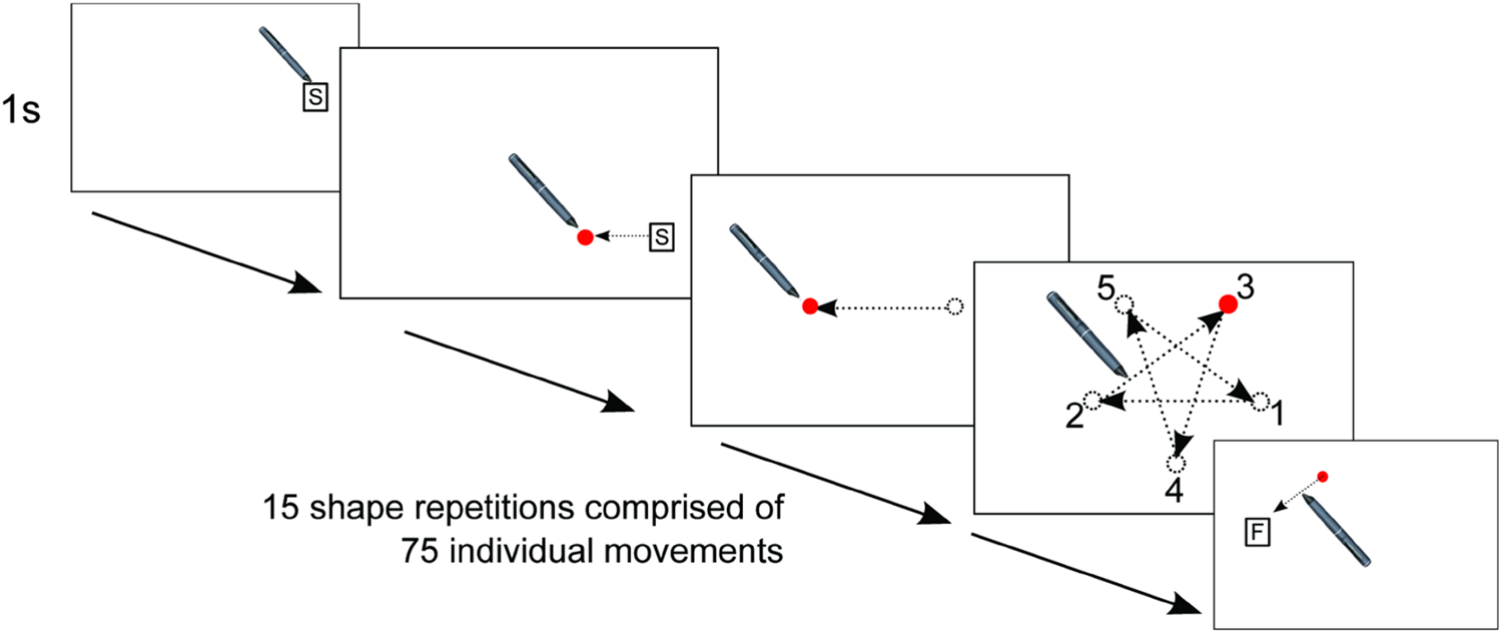
Schematic of the aiming task with *dotted arrows* to demonstrate the movements made by participants during the task

Participants first completed an aiming task with their preferred hand (see Flatters et al. 2014a for full task description). The task began by participants holding the stylus over a start position marker for 500 ms. This resulted in a green dot appearing; participants were instructed to move the stylus to this dot as quickly and as accurately as possible without lifting the stylus from the screen. Arrival at the target caused the dot to disappear and be replaced by another green dot in a different location- to which participants then aimed (see Fig. 4). This was repeated for a total of 75 trials after which a finish position marker appeared which terminated the task when contacted. Task performance was measured by determining the time taken to move between the green dots on each trial and then taking the average duration across all trials (see Flatters et al. 2014a for more details).

Participants then completed the number line task with their preferred hand, the procedure of which was identical to Experiment 1 (see Fig. 1). Movement time (MT) was computed for the aiming task. For the number line task, Reaction Time (RT), and Movement Time (MT) were calculated. The same exclusion criteria applied in Experiment 1 resulted in the removal of 8.49% trials.

## Results and discussion

### Data analysis

Reaction time and movement time data were used to examine the reproducibility of the MNL effects observed in Experiment 1.

### Number line task: RT

**Fig. 5:**
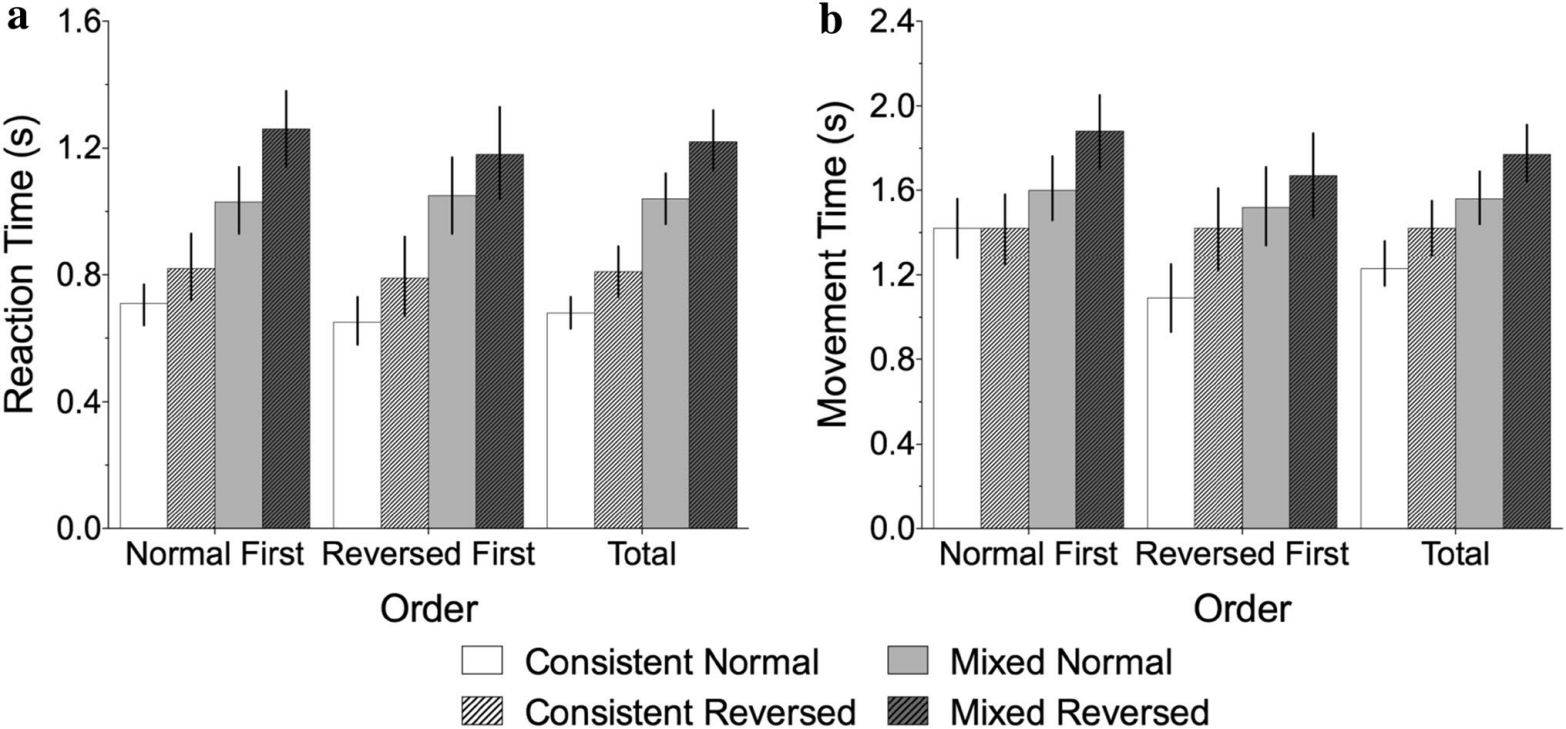
Reaction time (**a**) and movement time (**b**) data from Experiment 2

A mixed-design ANOVA with four trial types (normal, reversed, mixed normal and mixed reversed) and two conditions (Normal First, Reversed First) was conducted. The results showed the same patterns as observed in Experiment 1. A main effect of trial type was found, *F*(3, 135) = 117.48, *p* < .001, *η*_*p*_^*2*^ = .72; mixed trials showed slower reaction times than consistent trials. There was no effect of condition, *F*(1, 45) = .29, *p* = .593, *η*_*p*_^*2*^ = .01. There was also no interaction between trial type and condition, *F*(3, 135) = .747, *p* = .505, *η*_*p*_^*2*^ = .02; for both the consistent and mixed blocks, normal trials were responded to quicker than reversed trials (*p* < .001) (see Fig. 5a).

### Number line task: movement time

We found a significant effect of trial type on movement time (MT), *F*(3, 135) = 35.91, *p* < .001, *η*_*p*_^*2*^ = .44; mixed trials were slower than consistent trials demonstrating the increased information processing demands. Normal trials had a shorter duration in both the consistent trials (*p* < .05) and the mixed trials (*p* < .001); these effects can be seen in Fig. 5b. There was no main effect of condition, *F*(1, 45) = 2.195, *p* = .145, *η*_*p*_^*2*^ = .05; however, there was a significant interaction, *F*(3, 135) = 3.91, *p* < .05, *η*_*p*_^*2*^ = .08. In the consistent trials, the MNL effect was only observed in the Reversed First condition (*p* < .01); in the mixed trials, the MNL effect was observed irrelevant of condition.

In common with Experiment 1, we explored the extent to which the task affected both the cognitive operations and the movement execution by correlating the reaction time and movement time data across the participants. We found a significant correlation between the average reaction time and movement time within the mixed trials, *r*(47) = .844, *p* = .001.

### Number line task and distance error

Consistent with Experiment 1, a repeated measures ANOVA showed a significant effect of number type, *F*(7, 315) = 25.33, *p* < .001, *η*_*p*_^*2*^ = .36. Small numbers showed bigger distance errors than large numbers in all trial types (*p* < .05). In contrast to Experiment 1, a significant effect of trial type was observed, *F*(3, 138) = 5.125, *p* = .003, *η*_*p*_^*2*^ = .10. Larger distance errors were observed in the mixed trials, though this was only significant in the mixed normal trials (*p* < .05). These effects demonstrate that the increased cognitive load in the mixed blocks had some effect on accuracy within the number line task.

### Number line task performance and motor skill level

We next explored whether a reliable relationship existed between the number line task metrics and the aiming task measures (used as a proxy for motor ability). The motor skill performance measure was the average time that it took participants to move from one green dot to another in the aiming task. The results showed no significant correlations between motor skill level and the trials in the consistent blocks for reaction times (Normal, *r*(47) = .223, *p* = .131; Reversed, *r*(47) = .174, *p* = .243) and movement times (Normal, *r*(47) = .201, *p* = .176; Reversed, *r*(47) = .163, *p* = .275).

However, motor skill level (as measured by the aiming task) correlated significantly with reaction times to mixed normal trials, *r*(47) = .446, *p* = .002 and mixed reversed trials, *r*(47) = .311, *p* = .033. Furthermore, aiming correlated with movement times to mixed normal trials, *r*(47) = .439, *p* < .001 and mixed reversed trials, *r*(47) = .326, *p* = .025.

## General discussion

Experiments 1 and 2 suggest that mathematically literate adults use a default, culturally determined, mental number line (MNL) when undertaking a task that requires numbers to be mapped to a spatial location. In particular, participants consistently defaulted to a MNL when the cognitive demands of the task were increased. This was seen in both reaction times and movement times, across both experiments. There was also some evidence that adults used a MNL to complete less difficult tasks where the line orientation was the same within a block of trials, although this effect was weaker. The existence of a MNL is commensurate with other findings in the literature relating numbers and space, such as the SNARC effect and interval bisection in neglect patients (Dehaene et al. 1993; Dehaene 1997; Fischer and Shaki 2014).

 We were able to investigate the effect of prior exposure to a given spatial–numerical mapping by counterbalancing the order of the initial consistent trials. The responses within the mixed block should have been affected by the direction of the number line in the preceding block if the spatial–numerical mappings were altered by the recent exposure. Conversely, the responses within the mixed block should be unaffected by the preceding block direction if participants defaulted to culturally defined norms within our task. In the present experiment, we found that the default to a MNL within the mixed trials was not altered by recent exposure to an alternative spatial representation. This suggests that the cultural MNL representation was reasonably robust. This finding was not predicted from other research that has shown that SNARC effects can be altered by temporally recent spatial–numerical mappings (e.g., Fischer et al. 2010; Lindemann et al. 2007). There are various reasons that might explain why we did not see an effect of recent exposure. For example, the MLN effects may be highly flexible and thus influenced by the preceding trial. This would mean that any effect of the previous block was ‘washed’ out over the time taken to complete the mixed block. A reviewer suggested that it is also possible that the temporal resolution of our system (8 ms) prevented us detecting subtle effects. The fact that two blocks were run before the mixed block might also create complex interactions (and indeed there was evidence of learning affecting the responses in the consistent blocks). It seems clear that the effect of recent exposure is task specific and this might explain why we found a different effect to Fischer et al. (2010). The important point is that the participants in our experiment used a culturally defined MNL when performing our task and this allowed us to explore the relationship between sensorimotor ability and completing the numerical processing task.

The key finding from our experiments was an interactive relationship between numerical processing and the motor system. If numerical processing were encapsulated from sensorimotor control then there should have been no difference in the movements as a function of line direction. In this context, it is worth considering the design of a robotic system created to complete the task. One possible (and plausible) design would involve a computer controlling a robotic arm. The computer would undertake the numerical processing (converting the symbol to a proportion and determining the corresponding location on the line) before sending a control signal to the robotic arm (directing the arm to move to the calculated coordinates). In such an arrangement, the numerical processing is encapsulated from the control of the robotic motors. Thus, the fact that we found that movements altered as a function of line direction indicates that the numerical processing and motor systems operate synergistically in such tasks. We further explored this interdependency between numerical processing and action by investigating the relationship between participants’ sensorimotor ability and their performance on the number line task in Experiment 2. The results showed that there was no reliable relationship between performance on the aiming task and performance within the consistent blocks of the number line task. However, the level of motor skill was related to number line performance when the cognitive load was increased (in the mixed block). This provides evidence that numerical processing and motor processes are intrinsically linked. The fact that the consistent blocks showed no relationship with an individual’s motor abilities indicates that the numerical processing and motor processes are interacting in the mixed trials (i.e., the relationship with motor ability isn’t simply because the participants are responding to the task with an aiming movement). These observations are consistent with behavioural research which has demonstrated the influence of number on grip aperture and force production (Lindemann et al. 2007; Moretto and di Pellegrino 2008; Vierck and Kiesel 2010).

The mixed trials showed a clear advantage for ‘normal’ line direction and it was the mixed trials in Experiment 2 that revealed a correlation between the sensorimotor abilities of the participants and task performance. Notably, there was no correlation between sensorimotor ability and task performance in the easier blocks. It will be noted that the sensorimotor demands are identical between the ‘easy’ and ‘difficult’ conditions. Thus, what made the task demanding in the mixed trials was the requirement for numerical processing. This means that the only difference between the conditions related to the processing necessary to determine what trajectory the participant needed to follow—processing that needed to be completed before the participant could begin to move. But our data showed that both the time to complete the processing (reaction time) and the movement kinematics were predicted by the sensorimotor abilities of the individual. The fact that better sensorimotor ability improved performance on the task shows that these types of numerical processes are not independent of motor control—which supports the hypothesis outlined in the introduction. This finding is consistent with the large body of literature showing that spatial representations are used as cognitive tools in maths tasks and, consequently, that maths is intrinsically linked with a variety of sensorimotor processes. Nonetheless, it seems remarkable to find evidence that an individual’s ability to carry out numerical processing is related to how well they can control their hand movements. We suggest that the finding seems so remarkable because it is difficult to conceive of sensorimotor control being anything other than a simple system for outputting the complex cognitive operations that take place in the head.

Our findings are predicted by the neural substrates that underpin the representation of number in the primate brain. It is known that the prefrontal and the posterior parietal cortices are not only critical for sensorimotor control but also play a central role in number processing (Dehaene et al. 2003, 2004; Walsh 2003). There is also evidence to suggest that these areas play a role in the processing of information related to time. This organisation can explain the behavioural observation that number, space and time representations interfere with one another (Walsh 2003). Likewise, single-cell recordings and functional imaging data from humans suggest that representations of continuous and discrete quantities share a frontoparietal substrate (Tudusciuc and Nieder 2009).

It is important to emphasise that our findings do not suggest a unique relationship between sensorimotor control and numbers per se. van Dijk and Fias (2011) have suggested that the SNARC effect depends on the ordinal position of numbers in working memory rather than numerical size (see also Lindemann et al. 2007) and some authors have argued for the coexistence of separate mechanisms for processing ‘magnitude’ and ‘ordinality’ (Chen et al. 2014). Indeed, Krause et al. (2014) have demonstrated structural brain differences related to the mapping of numbers to space and the mapping of numbers to sensorimotor-related magnitude. The important point is that numbers are spatially represented in the brain and it is this shared representation between space and number that appears to drive the effect found within our study. In other words, numerical processing is a special case of a more general interaction between sensorimotor control and cognitive processes that rely on spatial representations (see, e.g., Gevers et al. 2003a, b) where sensorimotor control is not simply the output of cognitive operations, but influences those operations.

In conclusion, our results support the idea that spatial–numerical brain representations reflect a long-term exposure to culturally determined directional numerical organisation. It is notable that a myriad of everyday activities has both motor and numerical processing demands and our work suggests that a full understanding of these tasks requires a consideration of the interacting demands of these different neural systems. In this vein, we suggest that our experimental task might be usefully deployed to assess the nature of spatial–numerical associations and numerical representation in children, where problems in motor ability and number processing can often co-occur (Pieters et al. 2012). The present work leads to the strong prediction that there will be a relationship between sensorimotor ability and mathematical attainment in children—a prediction that can be tested empirically (see Simms et al. 2016).
